# Supplementary material for: A MicroRNA Next-Generation-Sequencing Discovery Assay (miND) for Genome-Scale Analysis and Absolute Quantitation of Circulating MicroRNA Biomarkers
Source: Int J Mol Sci. 2022 Jan 22;23(3):1226. doi: 10.3390/ijms23031226 (PMC8835905; doi:10.3390/ijms23031226)

Table S1. Overview of the selected small RNA sequencing kits

**Kit**	QIAseq miRNA	RealSeq-Biofluids Plasma/Serum miRNA	NEXTFLEX small RNA-seq kit v.3	CleanTag small RNA
**Company**	Qiagen	RealSeq Biosciences	Perkin Elmer	TriLink Biotechnologies
**Basic principle**	5’ and 3’ sequential adapter ligation	Single adapter ligation followed by circularization	5’ and 3’ sequential adapter ligation	5’ and 3’ sequential adapter ligation
**UMI**	+	-	+	-

Figure S1. Read counts distribution in miRXplore and plasma samples

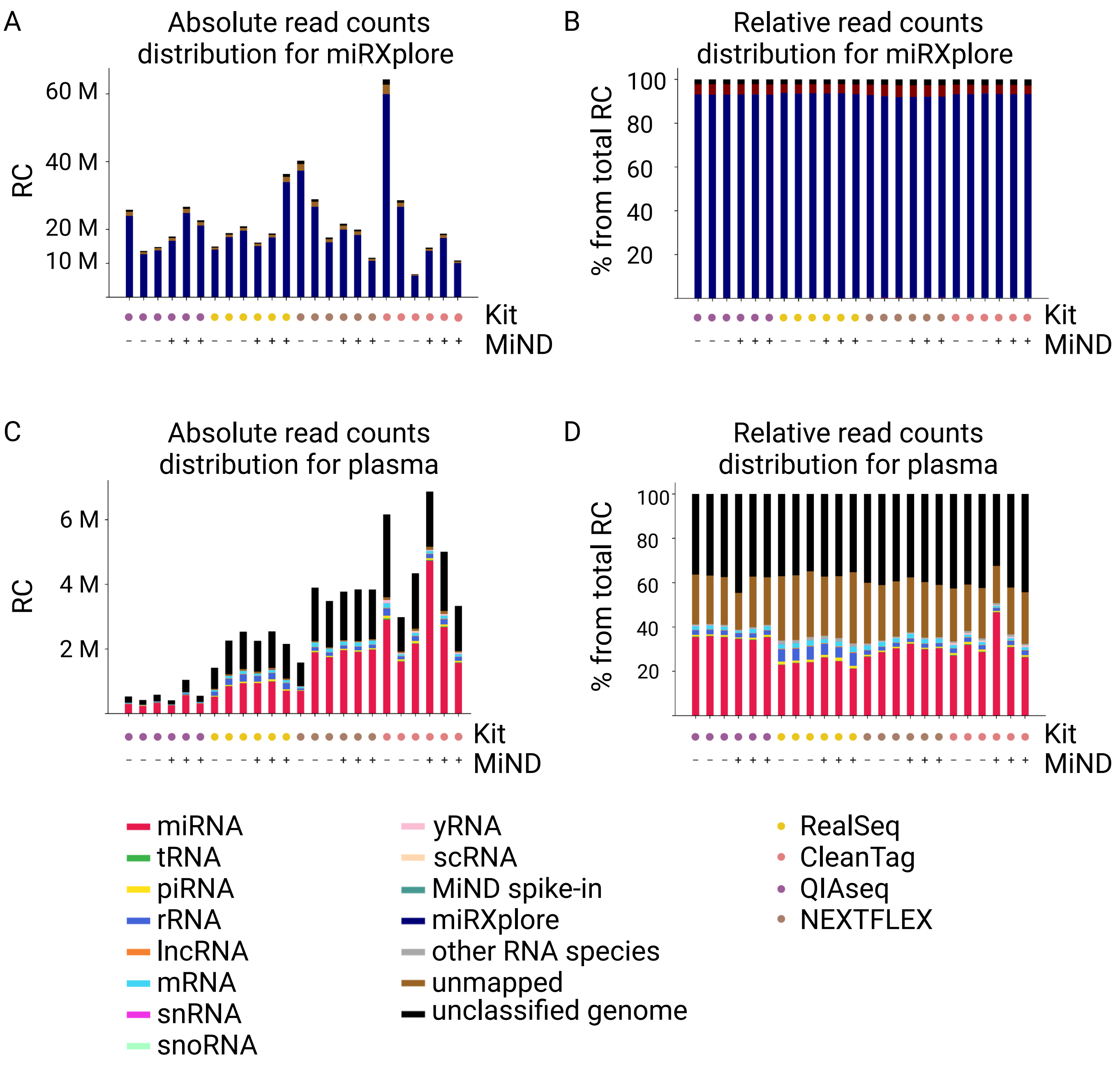

Figure S2. Number of distinct microRNAs

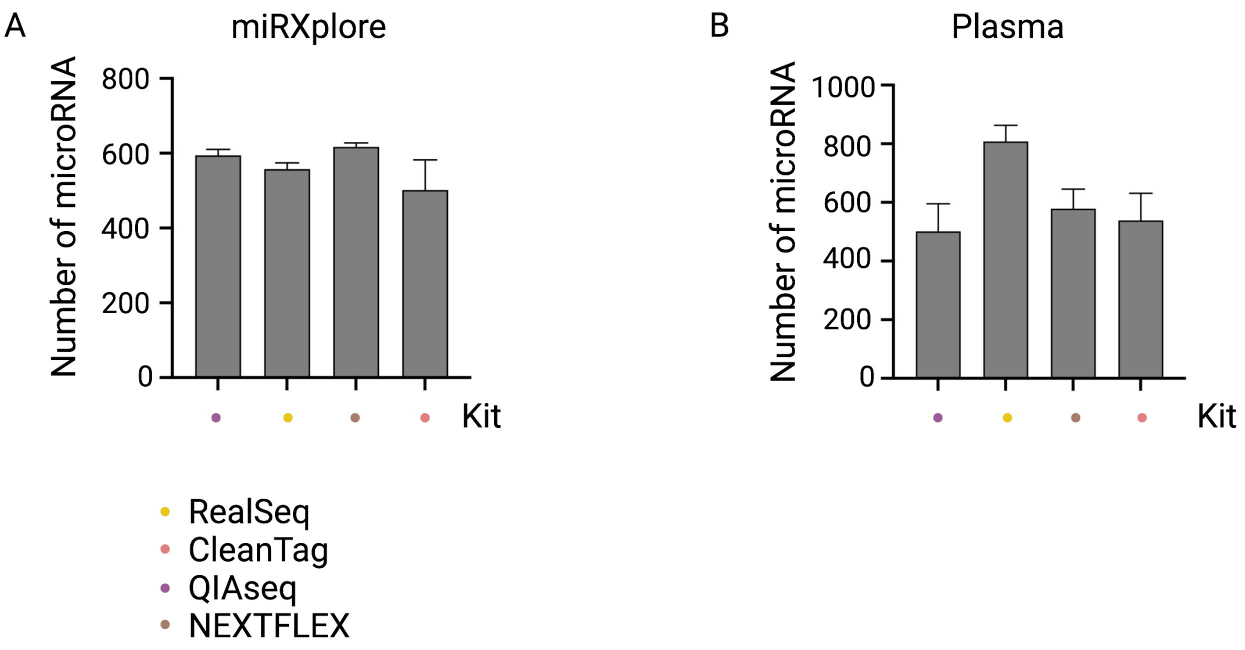

Figure S3. Performance of the selected small RNA sequencing protocols

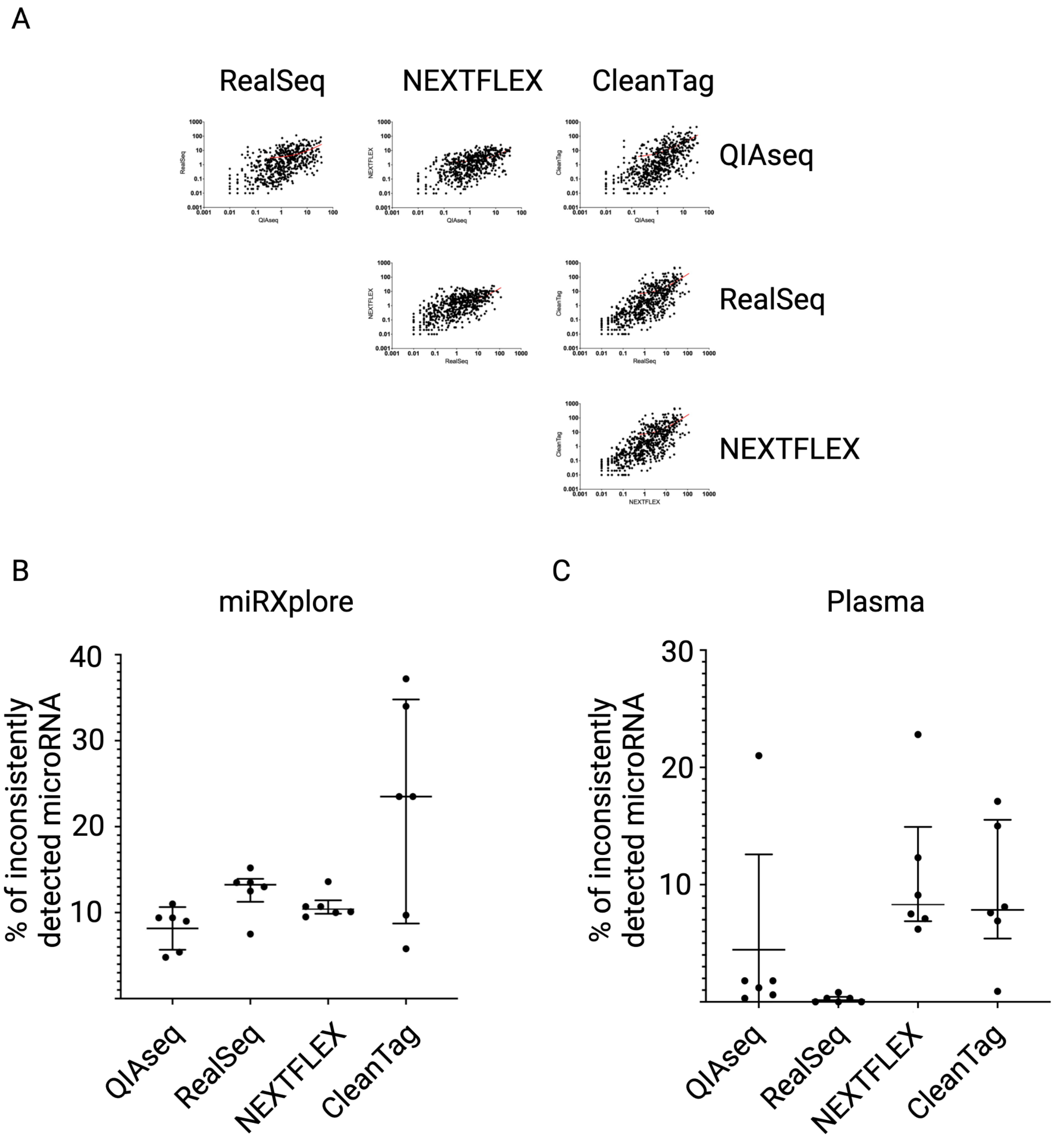

Figure S4. The 10 most and least abundant microRNAs

A

miRXplore - The most abundant microRNAs

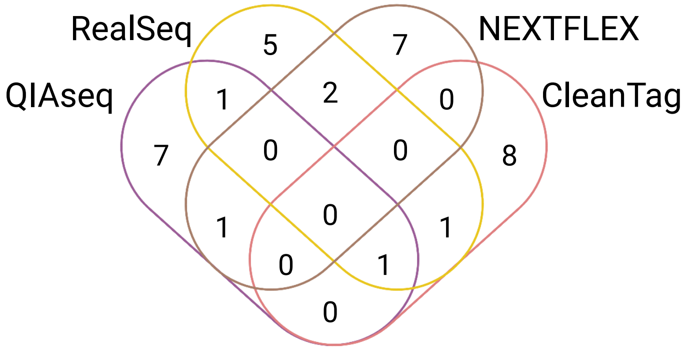

B

miRXplore - The least abundant microRNAs

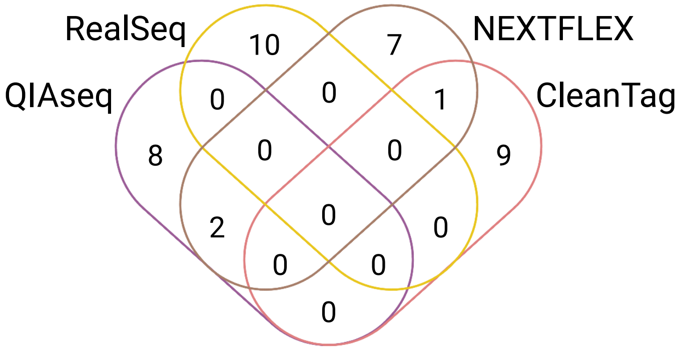

C

Plasma - The most abundant microRNAs

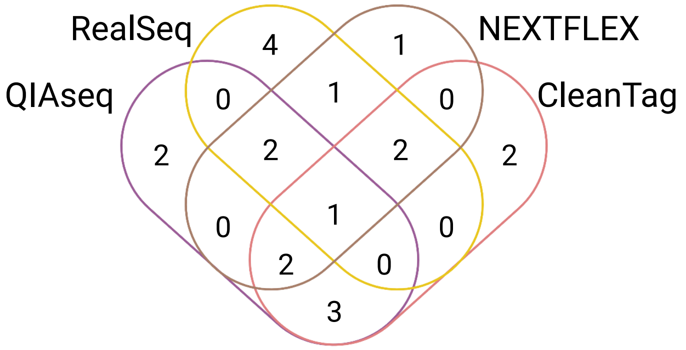

D

Plasma - The least abundant microRNAs

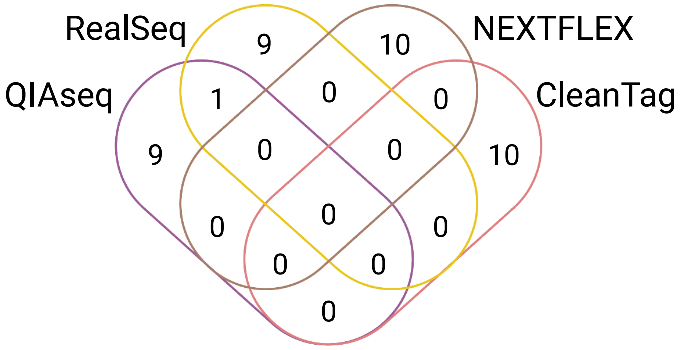

Figure S5. Scatter plots of the MiND spike-ins relative and absolute levels

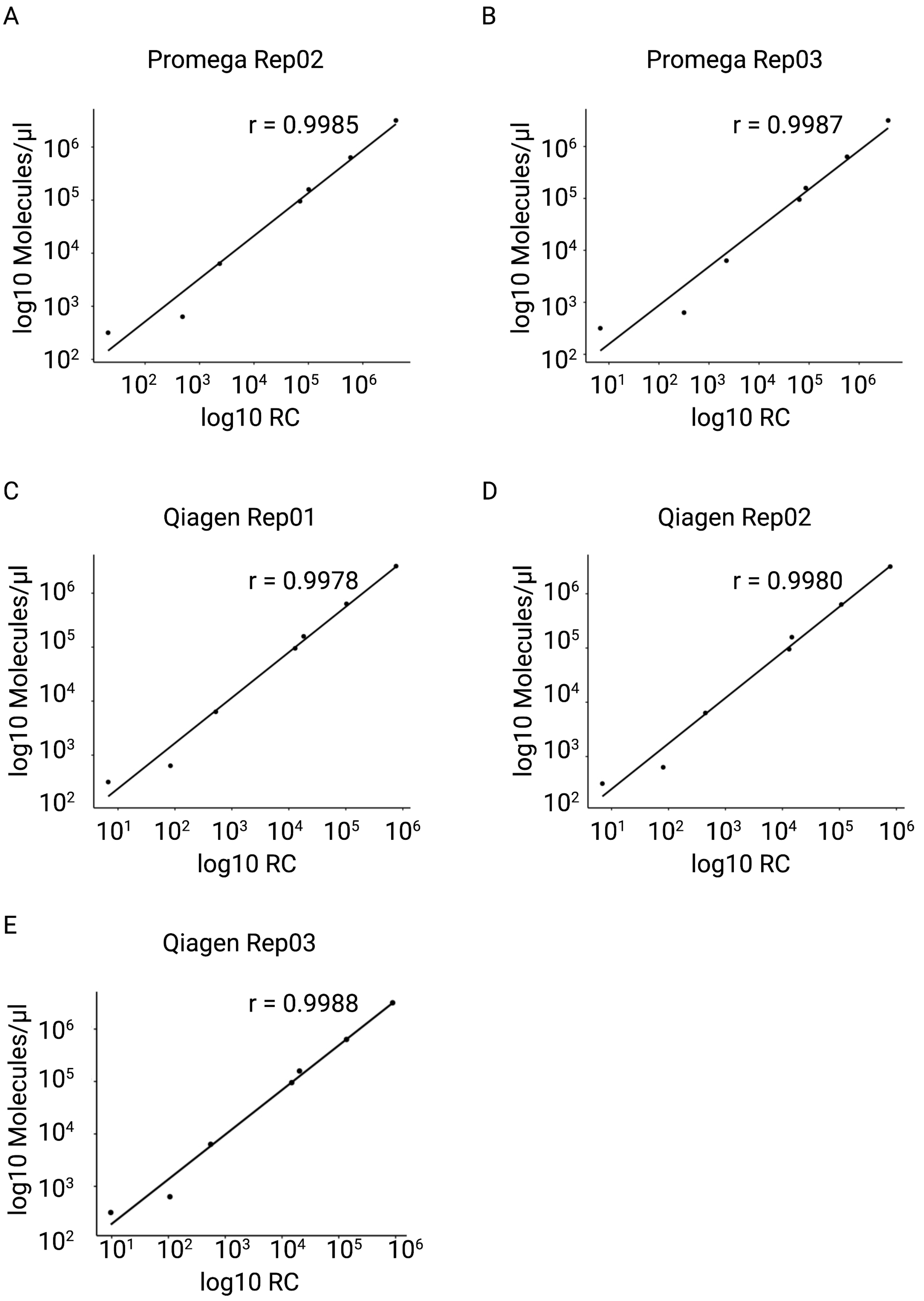

Figure S6. Optimisation of the small RNA NGS protocol

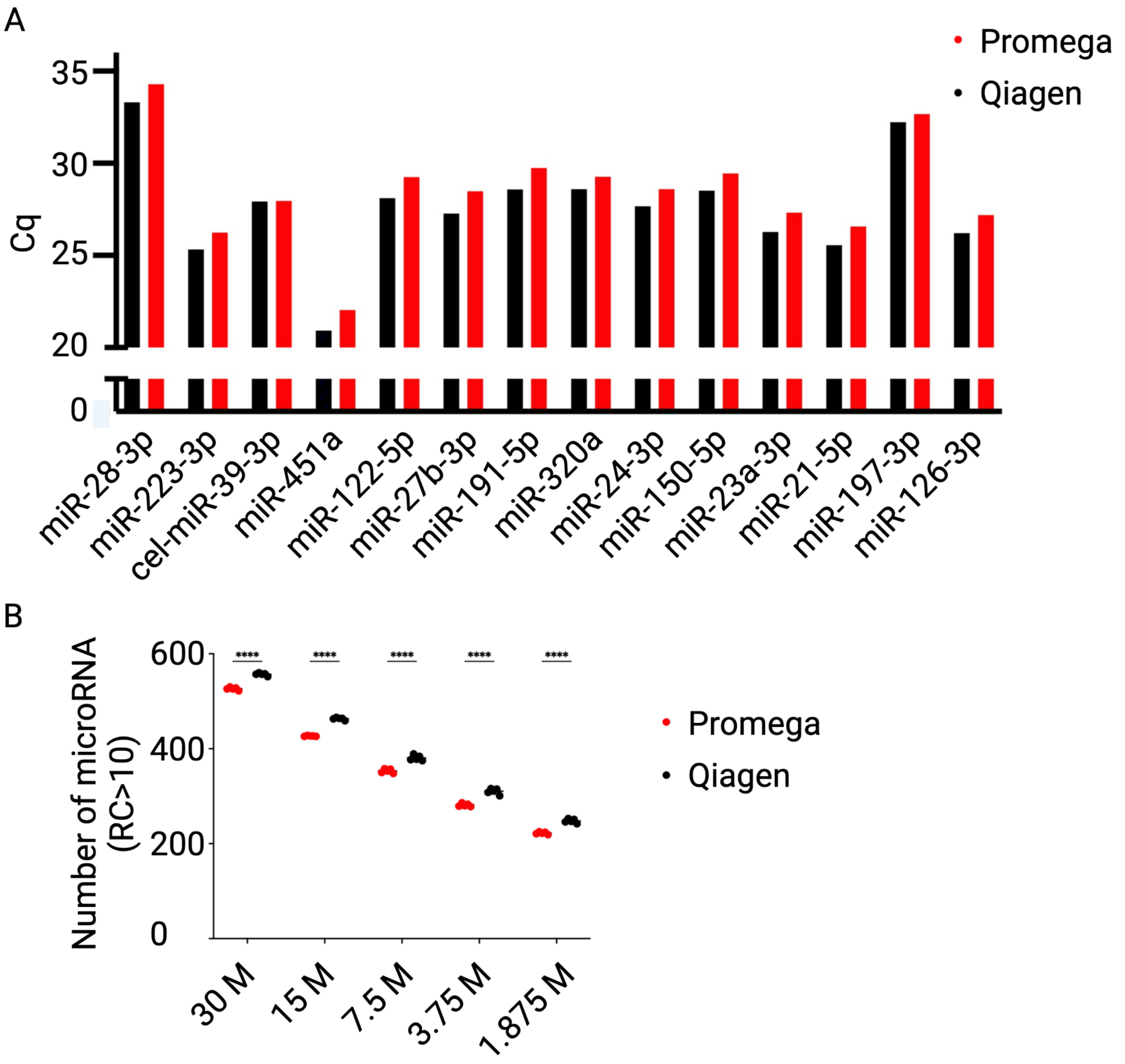

Figure S7. Impact of sequencing depth on number of detected microRNAs

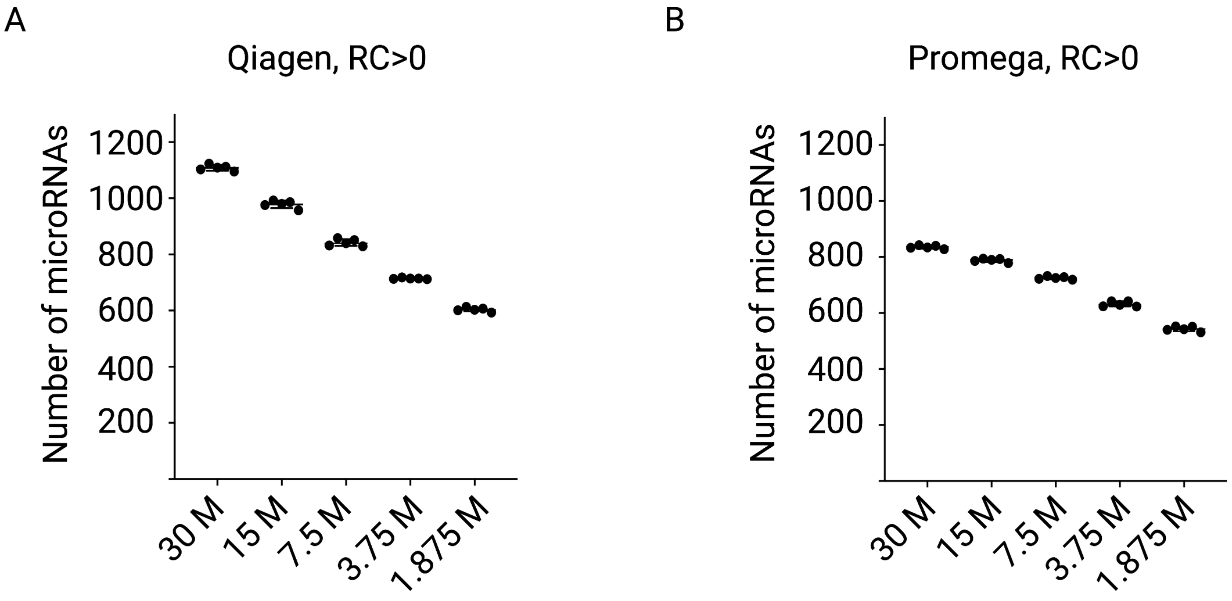

Figure S8. The MiND assay validation

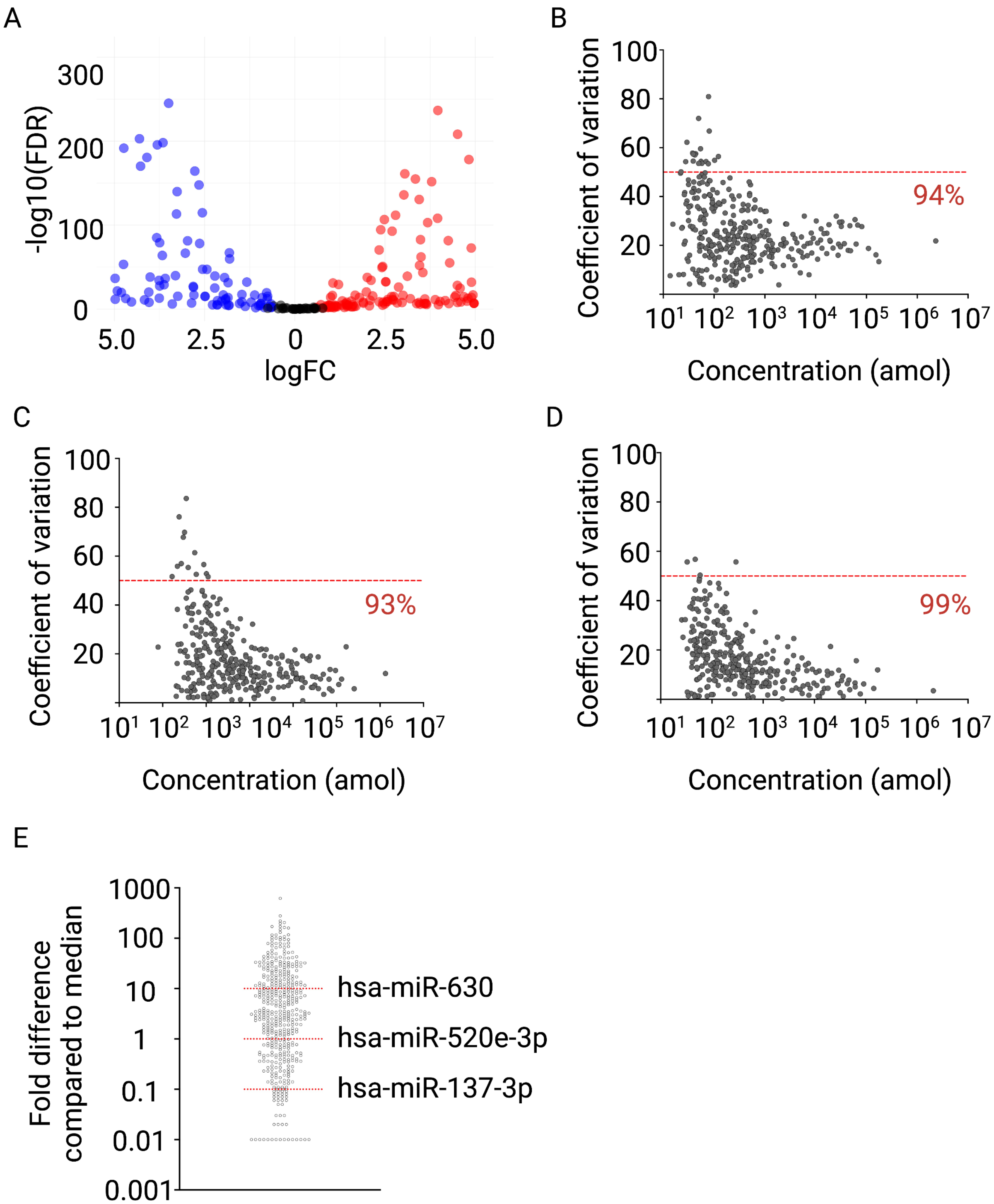

Supplement: Supplementary file 1 [file ijms-23-01226-s001.zip › ijms-1531533-supplementary-/Supplementary_Figures-Khamina-et-al.pdf]
